# Change of the State of the Natural Antioxidant Barrier of a Body and Psychological Parameters in Patients Aged above 60

**DOI:** 10.1155/2017/6568501

**Published:** 2017-12-19

**Authors:** Katarzyna Porzych, Beata Augustyńska, Marcin Porzych, Martyna Porzych, Emilia Mikołajewska, Daria Kupczyk, Rafał Bilski, Magdalena Żyła, Mirosława Szark-Eckardt, Kornelia Kędziora-Kornatowska

**Affiliations:** ^1^Department and Clinic of Geriatrics, Ludwik Rydygier Collegium Medicum in Bydgoszcz, Nicolaus Copernicus University, Toruń, Poland; ^2^Institute of Physical Education, Kazimierz Wielki University in Bydgoszcz, Bydgoszcz, Poland; ^3^Student Ludwik Rydygier Collegium Medicum in Bydgoszcz, Nicolaus Copernicus University, Toruń, Poland; ^4^Department of Physiotherapy, Ludwik Rydygier Collegium Medicum in Bydgoszcz, Nicolaus Copernicus University, Toruń, Poland; ^5^Neurocognitive Laboratory, Centre for Modern Interdisciplinary Technologies, Nicolaus Copernicus University, Toruń, Poland; ^6^Department of Biochemistry, Ludwik Rydygier Collegium Medicum in Bydgoszcz, Nicolaus Copernicus University, Toruń, Poland

## Abstract

**Background:**

The goal of this study is to assess the natural antioxidant barrier of the organism and selected psychological aspects of the aging process in patients above 60 years old.

**Methods:**

The study included a total of 52 patients aged above 60 (mean age 67 ± 3.4) and 32 healthy subjects (mean age 22 ± 3.4) as a control group. All patients underwent psychological assessment using Test of Attentional Performance version 2.3 (TAP 2.3, four subtests: alertness, cross-modal integration, neglect with central task, and working memory) and biochemical analysis of venous blood concerning values of the selected parameters of oxidative stress (HT, GSH, GPXOS, GPXRBC, GRRBC1, SODRBC1, MDARBC1, NO_2_^−^/NO_3_^−^, and CP).

**Results:**

Disorders of attention were observed mainly in elderly people, but an assumption that elderly people have developed more efficient ways of working memory use than younger people may be true. Results showed the reduced effectiveness of the body's natural antioxidant barrier in elderly people. Moderate positive and negative correlations among parameters of oxidative stress and psychological parameters were observed in the control group.

**Discussion:**

Intensification of the attention deficits and oxidative stress may be observed as one of the pathogenic factors of age-dependent diseases.

## 1. Introduction

Aging of the human body is a natural, complex, and long-lasting physiological process. It includes molecular changes both at the cellular level and at the level of the whole organism [1]. According to the World Health Organization, agedness begins at the age of 60. Nevertheless, the aging process is individualized and depends on ontogenetic, biological, and physical features, life experience, membership in the particular social group, practiced forms of activity, and so forth. Aforementioned interindividual variation during aging influences perception of life quality in elderly people, when life functions are weaker, and professional activity and social life are changing [2]. Although the aging process is inevitable, every health-oriented activity such as physical activity [3–6], cognitive activity [7, 8], optimal diet, and regular health care and rehabilitation may positively influence psychophysical condition, independence, living at home for the longest period possible [9], and thereby subjective feeling of life quality [10].

In the 1990s, three variants of aging were distinguished: normal (physiological), favorable (positive), and negative (pathological). Normal aging develops gradually; changes in an organism do not limit independent function instantly. Pathological aging covers diseases and sudden involutional changes, significantly limiting the independence of patients. Positive aging is still under research [11]. Hill describes happy aging as exploitation of every accessible resource within the optimal aging process. Physical predispositions, individual personal features, and social conditions may significantly affect this process. It requires great effort and a considerable amount of systematic work [12].

There are diverse definitions of happy aging and associated concepts [13–16]. Taking into consideration only changes in the human organism associated with aging, there may be observed developing morphological and biochemical changes at various levels: cellular, tissue, composition of the body, and body fluids. Reserves of the organism decrease, especially within respiration system, circulatory system, endocrine system, kidneys, and senses organs. Adaptability to physical and biological as far as psychosocial strains and homeostasis disorders decrease. Morbidity in patients aged more than 65 increases, especially in the area of endocrine disorders, diseases of cardiovascular system, tumors, and neurological diseases. Mortality increases with age. Changes presented above are regarded as inevitable but may develop gradually not disturbing the functioning of the organism at once. Usually, a happily aging patient is cheerful, willingly participates in the family and community life, and is mentally fit. And if ill, knowingly takes the challenge and tries to decrease the influence of the disease on the activities of daily living [17].

Scientists still look for causes of aging. One of them may be reactive oxygen species (ROS) and their role in the pathogenesis of neurodegenerative diseases, atherosclerosis, and damages of heart, brain, or lungs. Free-radical theory of aging (FRTA, Denham Harman, 1956) states that aging depends on enzymatic and nonenzymatic antioxidant mechanisms and amount of generated ROS. When oxidation reactions predominate in the organism, it develops oxidative stress [18]. The result of this process is a change in the structure and function of proteins, oxidation of cell membranes, and DNA damages [19]. Superoxide dismutase 1 (SOD1) and catalase (CAT) belong to the basic enzymatic components of the antioxidant system. SOD catalyzes the reaction of dismutation of superoxide radical anion to hydrogen peroxide, which is next decomposed by catalase to water and oxygen. Reduced glutathione (GSH) plays the main role in the enzymatic system, participating both directly and indirectly in antioxidant system, for example, reduction of hydrogen peroxide and lipid peroxides [20]. As a result of reactions above, glutathione disulfide, reduced by NADPH in a reaction catalyzed by glutathione reductase (GR), is formed. Antioxidant abilities of organisms also depend on content and activity of endogenous proteins such as ceruloplasmin [21].

A more detailed description of aging in two contexts: psychological and biochemical (antioxidant abilities of the organism) may be helpful in the assessment of the functioning of the aging organism.

### 1.1. Aim

This paper aims at the assessment of the natural antioxidant barrier of the organism and psychological assessment of the aging process in patients above 60 years old.

## 2. Material and Methods

### 2.1. Participants

**Figure 1:**
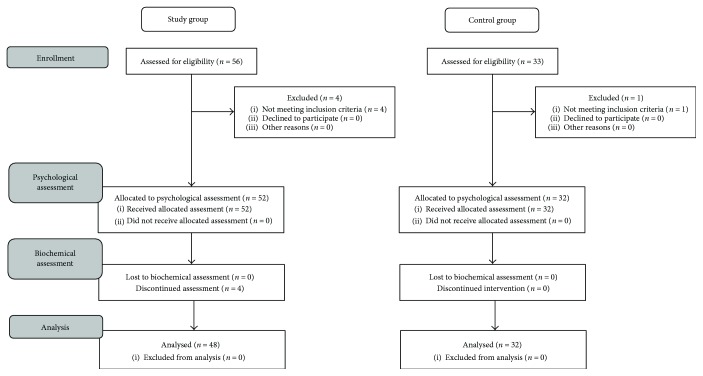
Patient's flow diagram.

The study group consisted of 48 patients of the nonpublic medical center “Bartodzieje” (26 women and 22 men, aged 60–80, mean age 67 ± 3.4 years). Most of the participants were intellectually, socially, and even professionally active (Figure 1).

Initial medical assessment and analysis of the patients' health records provided by physicians and psychologists allow determining whether the subjects were healthy.

The inclusion criteria were age ≥ 60 years, positively aging, lack of severe illnesses, with established history of physical activity, independence in activities of daily living, lack of discomfort within area of cognitive function, and lack of subjective deficits within everyday cognitive functioning.

The exclusion criteria were severe illnesses or injuries, cognitive function disorders, and history of physical activity as professional sportsmen or intensely trained. Authors are aware that antioxidant capacity can vary depending on the physical activity of participants; thus, authors provided a balance between the two groups.

The control group consisted of 32 healthy people (18 women and 15 men aged 22 ± 3.4), students or professionally active. Nineteen of them took part in a study concerning the function of an antioxidative barrier.

The inclusion criteria were age < 30 years, with established history of physical activity, lack of severe illnesses (e.g., diabetes mellitus) and injuries, independence in activities of daily living, lack of discomfort within area of cognitive function, and lack of subjective deficits within everyday cognitive functioning.

The exclusion criteria were severe illnesses or injuries, cognitive function disorders, and history of physical activity as professional sportsmen or intense trained.

Authors are aware that antioxidant capacity can vary depending on the physical activity of participants; thus, authors provided a balance within the two groups.

Authors did not obtain a measure of IQ, because study group consisted of people optimally, positively aging in well-being and without objective and subjective cognitive deficits. IQ measurement is applied in the case of lowering of cognitive function. From the other hand, assessment of participants' cognitive function showed a more detailed and clinically wider picture than IQ only. Preserved cognitive function during aging are associated with well-being in elderly and objective and subjective perception of well-being during aging.

This study was conducted in accordance with the Declaration of Helsinki and the guidelines for Good Clinical Practice (GCP). Freely given written informed consent was obtained from every patient before the study.

### 2.2. Methods

Attention was assessed using a computer application Test of Attentional Performance version 2.3 (TAP 2.3) by Peter Zimmermann and Bruno Fimm. The software consists of 13 subtests designed for assessment of various aspects of attention, including sensory integration, divided attention, attention shift, focusing, reaction to various stimuli, or working memory. The researcher may select an appropriate set of subtests according to the needs.

Every participant was instructed before the test. Participant of the test should respond (through pushing the key, one or both according to the task) to stimuli displayed on the screen. Pretests are available before every test to avoid misunderstanding. Every test was carried once in the study and control group.

Four subtests were applied in the study:

1. Alertness is the measurement of the simple reaction time to a cross appearing on the screen at randomly varying intervals where the subject responds as quickly as possible by pressing a key.
2. Cross-modal integration is the measurement of reaction to a combination of a preceding tone and a subsequent visual stimulus: high tone and an arrow pointing upward and low tone and an arrow pointing downward. Other combinations than above should cause lack of reaction.
3. Neglect with central task is the measurement of focus on central stimuli and simultaneous reaction to peripheral stimuli. Flicker stimulus is presented at different locations of the screen and varying intervals. The participant should press the key as quickly as possible when
  - stimulus “I0” is in the middle of the screen,
  - peripheral stimuli appear.
4. Working memory is the measurement of information flow and update in working memory: a sequence of numbers are displayed on the screen. The participant should press the key when the current number corresponds with the previous number.

The material for analysis was venous blood collected in an amount of approx. 8 ml of the antecubital vein into lithium heparin tubes and tubes without anticoagulant. Blood samples were collected at 8:00. Then, collected material was transported to the Department of Biochemistry of Nicolaus Copernicus University Collegium Medicum in Bydgoszcz. Tests were carried out on the same day, within approx. 1 hour of material collection.

The concentration of reduced glutathione (GSH) was assayed using the Beutler method [22]. The principle of this method is based on the reaction of reduction of the disulfide compound dithio-bis-2-nitrobenzoic acid (DTNB) by compounds containing sulfhydryl groups. In blood, free sulfhydryl groups unrelated to proteins are derived almost only from GSH. The product of the described reaction is a compound of yellow color. Color density was measured at 412 nm. In the calculations, the molar absorption coefficient was used, which, when attached to the mentioned wavelength, is equal to 13.6 (mol^−1^ × l × cm^−1^). The results were expressed in mmol/LRBC. The coefficient of variation for this method was 2.4%.

The activity of glutathione reductase (GR) in erythrocytes was assayed by a spectrophotometric measurement of NADP formation rate. NADP is the result of the reduction of glutathione oxidase in a reaction catalyzed by glutathione [23]. Change of absorbance was measured at wavelength 340 nm, and the result was expressed in U/g Hb. The variation coefficient for this method is 3.8%.

The activity of glutathione peroxidase (GPx) in erythrocytes was assayed by a two-stage Paglia and Valentine method [24]. In the first stage, GPx reacts with tert-butyl peroxide and reduced glutathione (GSH). The product of this reaction is glutathione disulfide (GSSG). The second stage involves the action of glutathione reductase (GR) reducing GSSG to GSH with the participation of NADPH + H^+^ as a regulator. NADPH oxidation results in a reduction in absorbance at a wavelength of 340 nm, which is measured spectrophotometrically. CGPx activity was calculated based on the loss of the reduced form of coenzyme in time (test Wartburg). In the calculations, millimolar absorption coefficient for NADPH at 340 nm, equal to 6.22 (mmol^−1^ × l × cm^−1^), was used. The results were expressed in U/g Hb, where 1*μ*mol oxidation of NADPH in one minute at *T* = 25°C was adopted as a unit of enzyme activity.

The coefficient of variation for this method was 2.9%.

Determination of glutathione S-transferase (GST) activity in RBCs was performed according to the method of Habig and Jakob [25]. In this method, there is a decrease in absorbance (which is measured at a wavelength of 340 nm) due to the formation of a conjugate of glutathione (GSH) with 1-chloro-2,4-dinitrobenzene (CDNB). The decrease in absorbance is proportional to the glutathione S-transferase activity. GST activity assay was carried out in the presence of phosphate buffer and CDNB. The results were expressed in nmol/CDNB-GSH/mg Hb/min.

Superoxide dismutase (SOD1) activity in RBCs was determined using the Misra and Fridovich method, which is based on the inhibition of adrenaline oxidation reaction by superoxide dismutase at pH 10.2 [26]. The increase in absorbance was measured at a wavelength of 480 nm. It is proportional to the increase in the concentration of oxidation products of adrenaline. The activity of SOD-1 was expressed in U/g Hb. The amount of enzyme which inhibits the oxidation of adrenaline 50% was adopted as a U unit. The coefficient of variation for this method is 6.3%.

The concentration of malondialdehyde (MDA) in the erythrocytes was determined by Placer et al.'s method, which is based on the reaction of a thiobarbituric acid and certain products of lipid peroxidation, mainly MDA, in an acidic environment and at elevated temperature [14]. This reaction produces a colored product, the color intensity of which was measured at a wavelength of 532 nm. In the calculations, the millimolar absorption coefficient of 156 (mmol^−1^ × l × cm^−1^) was used. The result was expressed in mmol/g Hb. The coefficient of variation for this method was 3.5%.

The concentration of nitric oxide was determined using the indirect method according to Marlett, determining the concentration of nitrate/nitrite in plasma. The method is based on the reaction between the nitrate anion and anion from N-(1-naphthyl)ethylenediamine, in the sulfanilic acid environment (Griess reaction) [27]. This reaction produced a colored complex whose absorbance is measured at a wavelength of 545 nm. It is directly proportional to the concentration of nitrates and nitrites in the studied sample. The result was expressed *μ*mol/L.

Ceruloplasmin oxidase activity was determined using the method of Ravin [28]. The principle of the method is based on oxidation of substrate p-phenyldiamine (PPD) by ceruloplasmin at a final purple-colored product. Absorbance measurement was made at a wavelength of 530 nm. This product is so-called “the principle of Bandrowski” (product formed from three molecules of the substrate). Results were expressed in international units.

### 2.3. Statistical Analysis

Statistical analysis was made using Statistica 12 software. The hypothesis of normal distribution was assessed by the Shapiro–Wilk test. Where available, results are expressed as the means ± standard deviation (SD) or median with minimal and maximal values. According to the needs, for data sets with normal distribution *t*-test was applied, and for the other data sets, Mann–Whitney *U* test was applied. Spearman's correlation coefficient was used to quantify the relationship between the parameters measured. The level of significance was set at *p* < 0.05.

## 3. Results and Discussion

### 3.1. Results

There were statistically significant differences observed between study group and control group in the “alertness” test. Reaction times were significantly shorter in the control group (both condition I and condition II, Table 1).

There were statistically significant differences observed between study group and control group in the “cross-modal integration” test, but not in the error-anticipation parameter. Reaction times to proper stimuli were significantly shorter in the control group as far as the number of errors (improper response, omission) in the same group (Table 2).

There were statistically significant differences observed between study group and control group in “neglect with central task” test. Participants from the study group responded slower to the stimuli both in the middle and peripheral area of the screen, as well as made more mistakes within the whole field of vision (Table 3).

There were no statistically significant differences observed between study group and control group in “working memory” test except errors—omitting. Contrary to the earlier subtests, participants from control group made significantly more errors than participants from the study group (Table 4).

The next part of the study was a statistical analysis of biochemical parameters (Table 5).

Mean concentrations of the reduced glutathione in erythrocytes was higher in the study group compared with the control group (*p* ≤ 0.001). The activity of glutathione peroxidase was lower in the study group compared with the control group (*p* ≤ 0.001). Also, the activity of this enzyme in erythrocytes was lower in the study group compared with the control group (*p* ≤ 0.001). There was also higher activity of the glutathione-S-transferase observed in the study group compared with the control group (*p* ≤ 0.001). The activity of superoxide dismutase (SOD) was significantly lower in the study group compared with the control group (*p* ≤ 0.001). The concentration of malondialdehyde (MDA) was significantly higher in the study group compared with the control group (*p* = 0.010). The concentration of nitric oxide in plasma (measured indirectly based on the concentration of nitrates/nitrites) was significantly higher in the study group compared with the control group. Other statistically significant differences between the study and the control group were not observed.

Moderate positive (CP, MDARBC1) and negative (GPXRBC, GSTRB, GRRBC1, and NO_2_^−^/NO_3_^−^) correlations among selected parameters of oxidative stress, and selected psychological parameters were observed in the control group (Table 6).

## 4. Discussion

Reactive oxygen species (ROS, free radicals) are formed within many biological processes. If they are liberated in physiological amounts, they play the role of mediators and adjusters providing the proper function of cells [29]. Influence of ROS on cells depends on their concentration and uptime. ROS should be produced under strict control of enzymatic and nonenzymatic antioxidant system. Imbalance of prooxidant and antioxidant processes in the cell (oxidative stress) can be observed if the immune system is inefficient and production of ROS is increased. Free radicals initiate processes of cell component oxidation resulting in cell damages and disturbances in the function of cells and tissues [30]. It is believed that destruction of cell components during aging is due to the increased (with aging) production of free radicals and oxidants [31]. Prevention of damages caused by free radical is fulfilled by the antioxidant system (thanks to both endogenous and exogenous compounds). Compounds above, enzymatic and nonenzymatic, inhibit production of free radicals [32].

Current knowledge concerning the influence of aging on superoxide dismutase (SOD) is ambiguous. There were species-dependent differences in this enzyme activity observed. It is believed that the differences mentioned above may be caused by the diverse speed of metabolism, including diverse speed of free radical production [33]. The highest activity was observed in these animal species in which processes associated with aging last the longest, and simultaneously speed of the metabolism is the smallest. The highest activity of SOD was observed in humans, then in the Asiatic elephant, chimpanzee, and horse [34]. But it is not a strict rule: the activity of SOD decreases with age in rats, but not in mice [24]. Lower activity of SOD was observed in people older than 60 years old compared with the control group. Age-dependent changes in SOD activity are influenced in animals by race, sex, and subcellular localization of this enzyme [35]. Reduced activity of SOD was measured in mitochondria of rat brain [36]. The age-dependent reduced activity of SOD is a result of its inactivation due to excessive production of hydrogen peroxide. Aging of human and animals may be accompanied by increase or decrease of concentration of reduced glutathione within various organs [37–39]. Higher values of this parameter were observed in the study group compared with the control group. Aging of human and animals may also be accompanied by an increase, no change, or decrease of concentration of glutathione reductase [35]. Lack of change of this parameter value was observed in the study group compared with the control group. There were sex-dependent differences observed in rats: increase and next decrease of glutathione reductase activity in aging males and decrease and next increase of glutathione reductase activity in aging females [40]. Decreased activity of glutathione peroxidase was observed in plasma of aging people [41]. This result was confirmed by own results. There was observed the decreased activity of this enzyme in erythrocytes of the study group compared to the control group. Antioxidant abilities also depend on the activity of endogenous antioxidant proteins, for example, ceruloplasmin which binding ions of transition metals decrease reactions of free radicals [21]. The concentration of ceruloplasmin increases with age both in healthy people and patients suffering from age-related diseases [42]. Any statistically significant differences were not observed in the reported study.

The result of the damages caused by oxidative stress may be measured indirectly through assessment of the concentration of 2-thiobarbituric acid analogues: the most commonly used is MDA [43]. Statistically significant increase of MDA concentration indicates a disturbance of the antioxidant barrier of the organism. A study by Mutlu-Türkoğlu et al. [44] showed a statistically significant increase of MDA in the serum of aging patients compared to adult people aged 21–40. Our results showed also a statistically significant increase of MDA concentration in the study group compared to the control group. Nitric oxide is also nonenzymatic antioxidant agent which plays an important role in the regulation of vessel wall homeostasis, picks up ROS, and defend against lipid peroxidation [45]. We measured nitrites/nitrates ratio to assess nitric oxide metabolism. Statistically significant increase of nitric oxide metabolism was observed in the blood of patients in the study group compared to the control group. Hayes et al. observed an increase of nitric oxide level in aging women practicing regular exercises. Thus, regular exercises in elderly people may positively influence the production of nitric oxide and, indirectly, the circulatory system. Another assessed parameter associated with disturbance of antioxidant barrier in erythrocytes was the activity of glutathione S-transferase (GST). GST physiologically catalyzes the reaction of glutathione disintegration to repair oxidized lipids [46]. Previous studies show a decrease in GST activity with age [47]. Our study does not show statistically significant differences between the study and the control group in this area.

Based on the analysis above, an assumption that increased activity of antioxidants can represent the protection of the organism against the oxidative stress may be true.

Aging has several dimensions: biological, social, and emotional. Aging causes a gradual decrease in fitness and limitation of the social participation. Alienation may deepen due to impairment of cognitive abilities. Alertness is associated with the general state of active sensing allowing the adequately quick reaction to various situations, including long-lasting readiness to quickly response to certain stimuli. It is necessary for road traffic where misleading or critical situations may signalize possible threats. The above ability determines the course of the other cognitive processes, for example, information encoding and storage. Our study showed shorter response times in the control group. Another assessment parameter, cross-modal integration is useful, for example, in road traffic. The sound of an approaching car and assessment of its distance based on view allows a threat's assessment. The efficient course of the multimodal perception is a key for direct attention and decision-making. Our study showed shorter response times in the control group. Test for neglecting with central task assessed peripheral vision: reaction to stimuli occurring outside the center of gaze (without focusing attention on them). People from the study group responded slower to stimuli within full field of vision and failed more often than the control group. Test for working memory showed a lack of differences between the study group and the control group, but a number of failed attempts due to ignored stimuli was significantly higher in the control group. This result may be explained by the assumption that elderly people developed more efficient ways of working on memory use than younger people.

Correlations among selected parameters of oxidative stress and selected psychological parameters observed in the control group may show a hidden link among them, probably strengthening with age and worsening oxidative stress parameters. There is a need for further studies in the aforementioned area.

There is large interindividual variation in the process of aging. Differences result from genetic factors, living conditions, work, lifestyle, and so forth. The efficiency of enzymatic and nonenzymatic antioxidative mechanisms decreases with age, while the amount of produced ROS increases. Intensification of the oxidative stress may be observed as one of the pathogenic factors of age-dependent diseases. Impaired function of the antioxidant enzymes and/or excessive production of ROS is observed. The progress of aging of the organism causes also increase in products of lipid peroxidation. An effective method for strengthening the immune system through stimulation of the antioxidant enzymes activity is a proper diet [48]. Longstanding physical training interferes with the prooxidative and antioxidative balance of the organism toward prooxidant processes. It causes increased activity of antioxidant enzymes [49].

Oxidative stress plays an important role in aging due to an age-dependent increase of cell damages caused by the activity of free radicals. High concentration of oxidative damages was observed in elderly people tissues. Causes of the age-dependent weakening of the antioxidant defense and repair are still not known. An assumption that disorder of the processes above can be the main cause of aging may be true. Thus, prevention of changes and strengthening the defense system would prolong health, fitness, cognitive abilities, and active lifestyle. Factors influencing both physiological and psychological success in happy aging may be the direction of further research.

## 5. Conclusions

Intensification of the attention deficits and oxidative stress may be observed as one of the pathogenic factors of age-dependent diseases. Factors influencing successful prevention of changes and strengthening the defense system may be a direction of further research.

## Figures and Tables

**Table 1:** Results of the “alertness” test (two conditions).

Parameter	Study group (*n* = 48)		Control group (*n* = 32)		*p* value
	Mean	SD	Mean	SD	
Condition I: without cue (ms)^∗^	304.6	51.8	228.4	31.7	≤0.001
Condition II: with cue (ms)^∗∗^	278.4	44.3	226.6	33.7	≤0.001

^∗^Measurement of reaction time for stimulus not preceded by a cue stimulus presented as warning tone; ^∗∗^measurement of reaction time for stimulus preceded by a cue stimulus presented as warning tone.

**Table 2:** Results of “cross-modal integration” test.

Parameter	Study group (*n* = 48)					Control group (*n* = 32)					*p* value
	Mean	SD	Median	Min	Max	Mean	SD	Median	Min	Max	
Reaction time (ms)	517.4	137.4	469.5	316	996	349.2	60.1	343.5	280	461	≤0.001
Improper response [−]^∗^	2.1	2.8	1	0	2	0.7	0.8	0	0	2	0.017
Terror anticipation [−]^∗∗^	0.4	1.2	0	0	8	0	0	0	0	0	ns
Error omission [−]^∗∗∗^	1.3	2.6	0	0	14	0.7	0.8	0	0	1	≤0.001

^∗^Response to the wrong stimulus; ^∗∗^response before pair of stimuli has appeared; ^∗∗∗^lack of response to the proper stimuli; ns: not significant.

**Table 3:** Results of “neglect with central task” test.

Parameter	Study group (*n* = 48)					Control group (*n* = 32)					*p* value
	Mean	SD	Median	Min	Max	Mean	SD	Median	Min	Max	
Reaction time, central point (ms)	513.6	111.3	504	394	834	458.9	87.3	541	338	621	0.001
Reaction time, peripherals (ms)^∗^	640.7	210.6	582	442	961	462.4	103.2	449.5	348	570	≤0.001
Reaction time, point (ms)^∗∗^	6.6	5.6	5	1	26	1	1.5	3	0	9	≤0.001
Error omission central point (−)^∗∗∗^	2.5	3.3	5	0	66	1	1.5	1	0	7	0.019
Error omission peripherals (−)^∗∗∗∗^	4.1	5.1	6	0	12	0.2	0.6	0	0	3	≤0.001

^∗^Area around central point (fixation point) with numbers; ^∗∗^response to the wrong stimulus; ^∗∗∗^lack of response to the proper stimuli; ^∗∗∗∗^lack of response to the proper stimuli.

**Table 4:** Results of “working memory” test.

Parameter	Study group (*n* = 48)					Control group (*n* = 32)					*p* value
	Mean	SD	Median	Min	Max	Mean	SD	Median	Min	Max	
Reaction time (ms)	541.7	117.5	503	375	958	548.8	159.2	511	351	1100	ns
Wrong response (−)^∗^	0.4	1	0	0	5	1.5	3.2	0	0	14	ns
Error—omitting (−)^∗∗^	0.4	1	0	0	8	1.8	1.7	1	0	6	≤0.001

^∗^Response to the wrong stimulus; ^∗∗^lack of response to the proper stimuli.

**Table 5:** Biochemical oxidative stress parameters.

Parameter	Study group (*n* = 48)		Control group (*n* = 19)		*p* value
	Mean	SD	Mean	SD	
HT	42.68	2.504	43.34	3.253	0.373
GSH (mmol/LRBC)	2.6021	0.34517	2.2079	0.16247	≤0.001
GPXOS (U/gHb)	201.156	37.9240	246.047	48.2888	≤0.001
GPXRBC (U/gHb)	14.836	2.5079	18.911	2.2573	≤0.001
GSTRB (nmol/mgHb/min)	3.146	0.5107	2.495	0.4236	≤0.001
GRRBC1 (U/gHb)	56.646	8.4139	55.147	11.2139	0.553
SODRBC1 (U/gHb)	2339.27	212.130	2805.26	184.771	≤0.001
MDARBC1 (mmol/gHb)	0.2731	0.02594	0.2529	0.03364	0.010
NO_2_^−^/NO_3_^−^ (*μ*mol/L)	1.9315	1.43506	0.8121	0.63482	0.002
CP (IU)	11229.556	213.4096	1340.358	542.4489	0.188

**Table 6:** Correlation between selected parameters in study group.

Parameter	“Alertness” test		“Cross-modal integration” test	“Neglect with central task” test		“Working memory” test
	Without cue	With cue		Reaction time, central point	Reaction time, peripherals	
HT	ns	ns	ns	ns	ns	ns
GSH	ns	ns	ns	ns	ns	ns
GPXOS	ns	ns	ns	ns	ns	ns
GPXRBC	ns	ns	ns	ns	−0.327 *p* = 0.029	
GSTRB	ns	ns	−0.246 *p* = 0.049	ns	ns	ns
GRRBC1	ns	ns	ns	−0.235 *p* = 0.041	−0.218 *p* = 0.047	ns
SODRBC1	ns	ns	ns	ns	ns	ns
MDARBC1	ns	ns	ns	ns	0.301 *p* = 0.045	ns
NO_2_^−^/NO_3_^−^	ns	ns	−0.292 *p* = 0.047	ns	ns	−0.265 *p* = 0.038
CP	0.288 *p* = 0.040	ns	0.312 *p* = 0.037	ns	0.355 *p* = 0.016	ns
